# Optimizing mature oocyte yield in IVF: clinical comparison of r-hFSH+r-hLH and HMG in women with a stimulation dosage of at least 300 IU of gonadotropins

**DOI:** 10.3389/fendo.2026.1696657

**Published:** 2026-01-29

**Authors:** Human Fatemi, Sahra Steinmacher, Laura Melado, Ibrahim ElKhatib, Laura Marqueta, Francisco Ruiz, Raquel Del Gallego, Erkan Kalafat, Barbara Lawrenz

**Affiliations:** 1 ART Fertility Clinics, Abu Dhabi, United Arab Emirates; 2 Department of Obstetrics and Gynecology, Koç University School of Medicine, Istanbul, Türkiye; 3 Reproductive Unit, UZ Gent, Gent, Belgium

**Keywords:** euploid count, r-hFSH+r-hLH, HMG, oocyte retrieval, ovarian stimulation

## Abstract

**Objective:**

To study the impact of luteinizing hormone (LH) in stimulation medication on the mature oocyte yield obtained in in women receiving ovarian stimulation (OS) with a dosage of at least 300 IU gonadotropins.

**Design:**

Retrospective cohort study (01/2016-05/2024).

**Setting:**

Tertiary assisted reproductive technology center.

**Patients:**

Women undergoing OS stimulation of at least 300 IU gonadotropins.

**Intervention:**

OS using r-hFSH+r-hLH or HMG as stimulation medication.

**Main outcome measure:**

Retrieved mature oocyte number.

**Results:**

A total of 1,286 patients (696 in the HMG group and 590 in the r-hFSH+r-hLH group) were included in the unmatched cohort. Before matching, the r-hFSH+r-hLH versus HMG-groups showed significant differences in AMH (0.8 vs 1.2ng/mL,p<0.001), starting doses (85.1% vs 70.1% on 450IU,p<0.001), and categorized BMI distribution (p=0.003). After propensity score matching (age, BMI, AMH, basal FSH, starting dose), 1052 cycles were analyzed with a 1:1 match ratio. In the matched cohort after further adjusting for confounders, r-hFSH+r-hLH use was associated with significantly higher collected oocyte count (IRR 1.10, 95% CI 1.03-1.17, p=0.004) and mature oocyte count (IRR 1.12, 95% CI 1.04-1.21, p=0.003) compared to HMG. The sensitivity analysis looking at the interaction of AMH with gonadotropin type showed the effect is mostly significant for those with normal ovarian reserve (AMH between 1.0-3.5ng/mL, IRR: 1.19, 95% CI: 1.07 to 1.33, P = 0.001) but not for those with low (≤1ng/mL, P = 0.180) or high ovarian reserve (>3.5ng/mL, P = 0.932). Analysis of maturation rates showed no significant effect of medication type (p=0.143). The euploid blastocyst count after stimulation in the matched cohort and HMG use was associated with a 22% lower yield compared to r-hFSH+r-hLH (IRR 0.78, 95% CI 0.66-0.93, p=0.006) after adjusting for AMH, basal FSH, female age, dose, body mass index.

**Conclusion:**

The use of r-hFSH+r-hLH is associated with a significantly higher mature oocyte and euploid blastocyst count compared to HMG and the effect was most pronounced in women with normal ovarian reserve.

## Introduction

Ovarian stimulation is a key component of assisted reproductive technology (ART), with the goal of optimizing follicular recruitment while maintaining safety and efficiency. Although the ideal number of oocytes for a successful outcome remains debated, recent studies have clearly shown that a higher oocyte yield, regardless of age, is associated with an increased likelihood of pregnancy (1).

The impact of different gonadotrophin preparations and different dosages used in ovarian stimulation on treatment outcome in women undergoing controlled ovarian hyperstimulation for ART has been widely debated. Recombinant human follicle-stimulating hormone (r-hFSH) is widely used to stimulate follicular growth, but the addition of luteinizing hormone (LH) activity remains a subject of debate, particularly in women with suboptimal ovarian response. There is some evidence that supplementing r-hFSH with recombinant human LH (r-hLH) during ART may have beneficial effects on outcomes in women with suboptimal response (2–4). Supplementation with LH may be beneficial owing to increased FSH receptor expression and growth, in addition to improved follicular recruitment and a reduced rate of granulosa cell apoptosis (5, 6).

Human menopausal gonadotropin (HMG), which exhibits both FSH and LH activity, is commonly used as an alternative ovarian stimulation strategy. However, its LH-like activity is primarily derived from human chorionic gonadotropin (hCG) rather than endogenous LH, with 75 IU of HMG containing 75 IU of FSH and 75 IU of LH-like activity that this is assessed by *In vivo* animal model, originating from approximately 10–11 IU of hCG (7). r-hFSH+r-hLH (Pergoveris^®^), with a fixed-ratio of 2:1 of FSH + LH, has been introduced to provide a consistent LH-to-FSH ratio, potentially enhancing follicular recruitment and oocyte quality. However, the efficacy of high-dose r-hFSH+r-hLH compared to HMG in women receiving a high daily dose between 300IU-450 IU to obtain a sufficient number of oocytes for insemination, remains inconclusive.

This study aims to compare the effectiveness of high-dose r-hFSH+r-hLH versus only HMG in women who received 300 IU or more to eliminate gonadotropin dose as a confounding factor and ensure nobody in the cohort was underdosed, by analysing key reproductive outcomes, such as the number of retrieved oocytes and maturity rate.

## Materials and methods

This retrospective cohort study included women with primary and/or secondary infertility due to female, male, mixed, or unknown causes, with a BMI ranging from 19 to less than 40 kg/m², who were pursuing IVF/ICSI treatment to achieve conception. A total of 1,286 patients aged 19–45 years treated at a tertiary referral ART centre between January 2016 and May 2024 received a starting gonadotropin dose of ≥300 IU when reduced ovarian reserve was anticipated based on AMH, AFC and/or elevated basal FSH, to minimize the risk of ovarian under-response, in accordance with previously published evidence (8). Eligible cycles were those with a recorded AMH value within one year prior to treatment, an endocrine profile at the initiation of stimulation, performed under a GnRH antagonist protocol, and that proceeded to oocyte retrieval. Patients with untreated endocrine disorders and with hormonal pretreatments or luteal phase stimulations were excluded from the analysis. Patients were stratified into groups based on body mass index (BMI) categories, age and the initial gonadotropin dose received. Data were extracted from the clinic’s medical record system (VRepro).

### Ovarian stimulation protocol

All patients underwent controlled ovarian stimulation using either r-hFSH+r-hLH (Pergoveris^®^, Merck Healthcare KgaA Germany) or HMG, starting on day 2 of the cycle. The dosage and type of gonadotropin was determined by the clinician based on patient characteristics and predefined institutional protocols. The allocation reflected evidence-based therapeutic criteria such as ovarian reserve profile and prior response history, rather than individual clinician preference.

A GnRH antagonist was administered on day 5 of stimulation to prevent premature LH surges. Serum FSH levels were measured at baseline (before stimulation initiation) and on the day of final oocyte maturation. Final oocyte maturation was achieved by administration of hCG (5000/10000IU° and 0.3 mg of GnRH agonist (triptorelin), as dual trigger as soon as 2 leading follicles reached ≥17mm in diameter or at a smaller follicle size in case of very poor responder (9). Oocyte retrieval was carried out 34–36 h after. Oocyte retrieval procedure was performed under ultrasound guidance and all accessible follicles, independent of their size were aspirated. The follicular fluid was inspected for the presence of cumulus-oocyte-complexes. Mature oocyte (MII - oocytes at the metaphase II stage) count was noted in the medical record system.

### Statistical methods

Variables were presented as median and interquartile range for continuous variables and count and percentage of the total for categorical variables. Baseline characteristics and stimulation outcomes between the HMG and r-hFSH+r-hLH groups were compared using Mann-Whitney U test for continuous variables and chi-squared test for categorical variables. To account for potential confounding, we performed optimal pair matching with optimal matching algorithm. This approach minimizes the sum of absolute pairwise distances in the matched sample, reducing the likelihood of extreme within-pair distances and creating well-balanced groups without specifying matching order (10).

Covariate balance before and after matching was assessed visually using standardized mean differences and matched cohort density plots for both treatment groups. We implemented a doubly robust estimation approach by combining matching with regression adjustment in the matched cohort. This method provides consistent estimation of treatment effects even if either the matching process or the regression model is mis specified, offering additional protection against residual confounding (11). Mixed-effects negative binomial regression was used to analyze factors associated with collected oocyte count (COC) and mature oocyte count (MII), while mixed-effects logit-binomial regression was employed for maturation rates (MII per COC). Models included medication group, AMH, basal FSH, starting dose, age group, BMI category, and total stimulation days as fixed effects and patient identifiers as random intercepts for the repeated cycles of the same couple. Results are presented as incidence rate ratios (IRR) or odds ratios (OR) with 95% confidence intervals. Statistical significance was set at p<0.05. All analyses were performed using R statistical software (v.4.2.0).

### Ethics approval

The study was approved by the local ethics committee (REFA120-2312-ABU-027 and REFA120a-2312-ABU-027).

## Results

A total of 1,286 patients (696 in the HMG group and 590 in the r-hFSH+r-hLH group) were included in the unmatched cohort. Baseline characteristics showed significant differences between groups (**Table 1**). Patients in the r-hFSH+r-hLH group were older (median 40.0 vs. 39.0 years, p=0.001) with a higher proportion of patients in the >40 age categories (48.3% vs. 37.5%). r-hFSH+r-hLH patients had lower AMH levels (median 0.8 vs. 1.2 ng/mL, p<0.001), higher basal FSH (median 8.1 vs. 7.7 IU/L, p=0.005), and were more likely to receive the highest starting dose of 450 IU (85.1% vs. 70.1%, p<0.001). After optimal pair matching, 526 patients from each group were included in the matched cohort. Most baseline characteristics were successfully balanced (**Table 2**; **Supplementary Figures S1**, **S2**), with no significant differences in age, BMI, AMH, basal estradiol, or starting dose. Basal FSH levels were also comparable between groups in the matched cohort (median 8.1 vs. 8.0 IU/L, p=0.797).

**Table 1:** Baseline characteristics and stimulation outcomes in patients treated with HMG and r-hFSH+r-hLH, unmatched cohort.

Variable	Levels	HMG (n=696)	r-hFSH+r-hLH (n=590)	p*
Age (in years)	Median (IQR)	39.0 (36.0 to 42.0)	40.0 (37.0 to 43.0)	0.001
Age (SART categories)	<35	133 (19.1)	85 (14.4)	0.002
	35-37	122 (17.5)	88 (14.9)	
	38-40	180 (25.9)	132 (22.4)	
	41-42	96 (13.8)	116 (19.7)	
	>42	165 (23.7)	169 (28.6)	
BMI (kg/m^2^)	Median (IQR)	28.3 (25.0 to 31.5)	28.4 (25.9 to 31.3)	0.626
BMI (WHO categories)	≤25 kg/m^2^	176 (25.3)	114 (19.3)	0.003
	>25 to ≤30 kg/m^2^	258 (37.1)	270 (45.8)	
	≥30kg/m^2^	262 (37.6)	206 (34.9)	
AMH (ng/ml)	Median (IQR)	1.2 (0.6 to 2.0)	0.8 (0.4 to 1.7)	<0.001
Stimulation days (n)	Median (IQR)	9.0 (8.0 to 10.0)	10.0 (8.0 to 11.0)	0.689
Gonadotropin start dosage	300 IU	142 (20.4)	61 (10.3)	<0.001
	375 IU	66 (9.5)	27 (4.6)	
	450 IU	488 (70.1)	502 (85.1)	
FSH basal (IU)	Median (IQR)	7.7 (6.2 to 9.7)	8.1 (6.4 to 10.4)	0.005
E2 basal (pg/ml)	Median (IQR)	38.5 (28.9 to 51.7)	38.7 (27.1 to 51.6)	0.552
FSH at trigger (IU)	Median (IQR)	24.2 (19.9 to 30.3)	23.1 (19.3 to 28.3)	0.002
E2 at trigger (pg/ml)	Median (IQR)	1423.0 (790.8 to 2329.0)	1097.0 (509.9 to 2004.0)	<0.001
P4 at trigger (ng/ml)	Median (IQR)	0.4 (0.2 to 0.7)	0.4 (0.2 to 0.7)	0.346
COC (n)	Median (IQR)	7.0 (3.0 to 11.0)	6.0 (3.0 to 11.0)	0.111
MII (n)	Median (IQR)	5.0 (3.0 to 9.0)	5.0 (2.0 to 8.0)	0.061
Maturation rate (%)	Median (IQR)	85.7 (70.0 to 100.0)	85.7 (66.7 to 100.0)	0.226

*Mann-Whitney-U test or chi-squared test.

AMH, Anti-Mullerian-Hormone; BMI, Body Mass Index; SART, Society for Assisted Reproductive Technology; E2, estradiol; P4, progesterone; COC, Cumulus oocyte complex; MII, metaphase II oocyte; HMG, human menopausal gonadotropin; IQR, interquartile range.

**Table 2:** Baseline characteristics and stimulation outcomes in patients treated with HMG and r-hFSH+r-hLH, matched cohort.

Variable	Llevels	HMG (n=526)	r-hFSH+r-hLH (n=526)	p
Age (in years)	Median (IQR)	40.0 (37.0 to 43.0)	40.0 (37.0 to 43.0)	0.275
Age (SART categories)	<35	75 (14.3)	71 (13.5)	0.469
	35-37	87 (16.5)	84 (16.0)	
	38-40	140 (26.6)	120 (22.8)	
	41-42	80 (15.2)	96 (18.3)	
	>42	144 (27.4)	155 (29.5)	
BMI (kg/m^2^)	Median (IQR)	28.4 (25.2 to 31.4)	28.4 (25.8 to 31.3)	0.980
BMI (WHO categories)	≤25 kg/m^2^	123 (23.4)	107 (20.3)	0.143
	>25 to ≤30 kg/m^2^	203 (38.6)	234 (44.5)	
	≥30kg/m^2^	200 (38.0)	185 (35.2)	
AMH (ng/ml)	Median (IQR)	0.9 (0.5 to 1.6)	0.8 (0.4 to 1.6)	0.099
AMH categories	≤1.1	308 (58.6)	321 (61.0)	0.553
	>1.1 to ≤3.5	202 (38.4)	186 (35.4)	
	>3.5	16 (3.0)	19 (3.6)	
Stimulation days (n)	Median (IQR)	9.0 (8.0 to 10.0)	10.0 (8.0 to 11.0)	0.173
Gonadotropin start dosage	300 IU	51 (9.7)	51 (9.7)	1.000
	375 IU	22 (4.2)	22 (4.2)	
	450 IU	453 (86.1)	453 (86.1)	
FSH basal (IU)	Median (IQR)	8.0 (6.4 to 10.3)	8.1 (6.4 to 10.2)	0.797
E2 basal (pg/ml)	Median (IQR)	38.1 (28.4 to 52.5)	38.3 (27.0 to 51.0)	0.438
FSH at trigger (IU)	Median (IQR)	25.7 (21.2 to 31.3)	23.2 (19.4 to 28.4)	<0.001
E2 at trigger (pg/ml)	Median (IQR)	1137.0 (639.2 to 1915.5)	1107.0 (532.8 to 1969.0)	0.404
P4 at trigger (ng/ml)	Median (IQR)	0.4 (0.2 to 0.6)	0.4 (0.2 to 0.7)	0.209
COC (n)	Median (IQR)	5.0 (3.0 to 9.0)	6.0 (3.0 to 11.0)	0.023
MII (n)	Median (IQR)	4.0 (2.0 to 7.0)	5.0 (2.0 to 8.0)	0.082
Maturation rate (%)	Median (IQR)	85.7 (70.4 to 100.0)	85.7 (66.7 to 100.0)	0.101

AMH, Anti-Mullerian-Hormone; BMI, Body Mass Index; SART, Society for Assisted Reproductive Technology; E2, estradiol; P4, progesterone; COC, Cumulus oocyte complex; MII, metaphase II oocyte; HMG, human menopausal gonadotropin; IQR, interquartile range.

**Figure 1:**
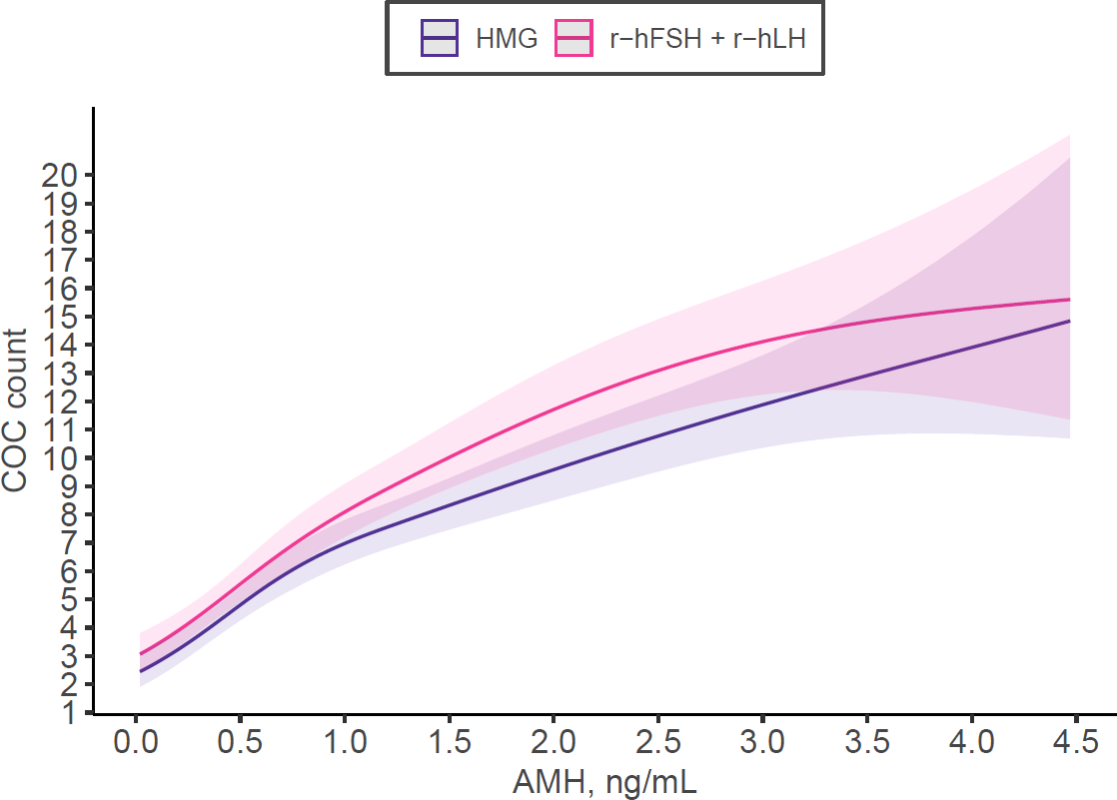
Number of Cumulus-oocyte-complexes (COC) retrieved depending on Anti-Muellerian-Hormone (AMH) and stimulation medication used according to multivariable regression model adjusting for AMH, basal FSH, starting dose, female age, BMI, total stimulation duration in the matched cohort.

In the matched cohort, the r-hFSH+r-hLH group demonstrated significantly lower FSH levels at trigger (median 23.2 vs. 25.7 IU/L, p<0.001), while estradiol and progesterone levels at trigger were comparable between groups (p=0.404 and p=0.209, respectively). Mixed-effects multivariable negative binomial regression showed that r-hFSH+r-hLH was associated with a 14% higher number of oocytes retrieved compared with HMG (IRR 1.14, 95% CI 1.07–1.21; p<0.001), after adjustment for confounders (**Table 3**; **Figure 1**). No significant association between stimulation regimen and oocyte maturation rate was observed (OR 0.92, 95% CI 0.82–1.03; p=0.170). Higher AMH levels were independently associated with slightly lower maturation rates (OR 0.92, 95% CI 0.84–1.00; p=0.044) (**Table 4**).

**Table 3:** Mixed-effects negative binomial regression analysis for factors associated with collected oocyte yield, matched cohort.

Variables	Incidence Rate Ratios	95% CI	P*
Gonadotropin type			
• HMG	Reference		
• r-hFSH+r-hLH	1.14	1.07 – 1.21	**<0.001**
AMH	1.18	1.14 – 1.22	**<0.001**
FSH basal	0.95	0.94 – 0.96	**<0.001**
AFC basal	1.07	1.06 – 1.08	**<0.001**
Gonadotropin starting dose			
• [300 IU]	Reference		
• [375 IU]	1.16	1.00 – 1.34	0.051
• [450 IU]	1.17	1.05 – 1.30	**0.005**
Female age, SART			
• <35			
• 35-37	0.99	0.89 – 1.10	0.846
• 38-40	0.88	0.80 – 0.96	**0.007**
• 41-42	0.82	0.74 – 0.91	**<0.001**
• >42	0.82	0.74 – 0.90	**<0.001**
BMI category			
• ≤25 kg/m^2^			
• >25 to ≤30 kg/m^2^	0.97	0.90 – 1.05	0.494
• ≥30kg/m^2^	0.99	0.92 – 1.08	0.884
Stim days (n)	1.09	1.07 – 1.11	**<0.001**

*Mixed effects multivariable negative binomial regression.

AMH, Anti-Mullerian-Hormone; BMI, Body Mass Index; SART, Society for Assisted Reproductive Technology; Gn, Gonadotropin; E2, estradiol; P4, progesterone; COC, Cumulus oocyte complex; MII, metaphase II oocyte; HMG, human menopausal gonadotropin.

Bold values are significant.

**Table 4:** Mixed-effects logit-binomial regression analysis for factors associated with maturation rates, matched cohort.

Variables	Odds ratios	95% CI	P*
Gonadotropin type			
• HMG	Reference		
• r-hFSH+r-hLH	0.92	0.82 – 1.03	0.170
AMH	0.89	0.84 – 0.95	**<0.001**
FSH basal	1.02	1.00 – 1.04	0.090
AFC basal	1.03	1.01 – 1.04	**<0.001**
Gonadotropin starting dose			
• [300 IU]	Reference		
• [375 IU]	1.21	0.95 – 1.55	0.133
• [450 IU]	1.12	0.94 – 1.34	0.216
Female age, SART			
• <35	Reference		
• 35-37	1.00	0.84 – 1.20	0.958
• 38-40	1.12	0.95 – 1.33	0.183
• 41-42	1.28	1.06 – 1.56	**0.012**
• >42	1.30	1.08 – 1.56	**0.006**
BMI category			
• ≤25 kg/m^2^	Reference		
• >25 to ≤30 kg/m^2^	1.01	0.87 – 1.18	0.865
• ≥30kg/m^2^	0.87	0.75 – 1.01	0.064
Stim days (n)	1.04	1.00 – 1.08	**0.027**

*Mixed effects multivariable logistic regression analysis.

AMH, Anti-Mullerian-Hormone; BMI, Body Mass Index; SART, Society for Assisted Reproductive Technology; Gn, Gonadotropin; E2, estradiol; P4, progesterone; COC, Cumulus oocyte complex; MII, metaphase II oocyte; HMG, human menopausal gonadotropin.

Bold values are significant.

**Figure 2:**
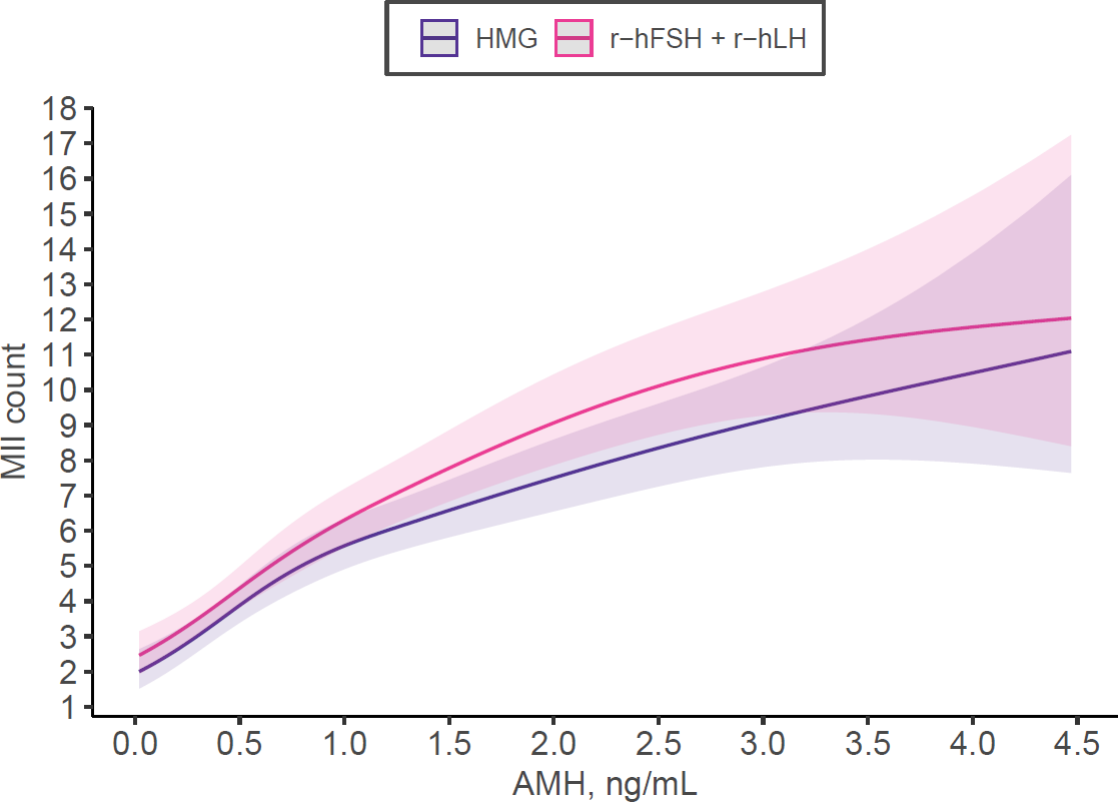
Number of mature oocytes (MII) retrieved depending on Anti-Muellerian-Hormone (AMH) and stimulation medication used according to multivariable regression model adjusting for AMH, basal FSH, starting dose, female age, BMI, total stimulation duration in the matched cohort.

**Figure 3:**
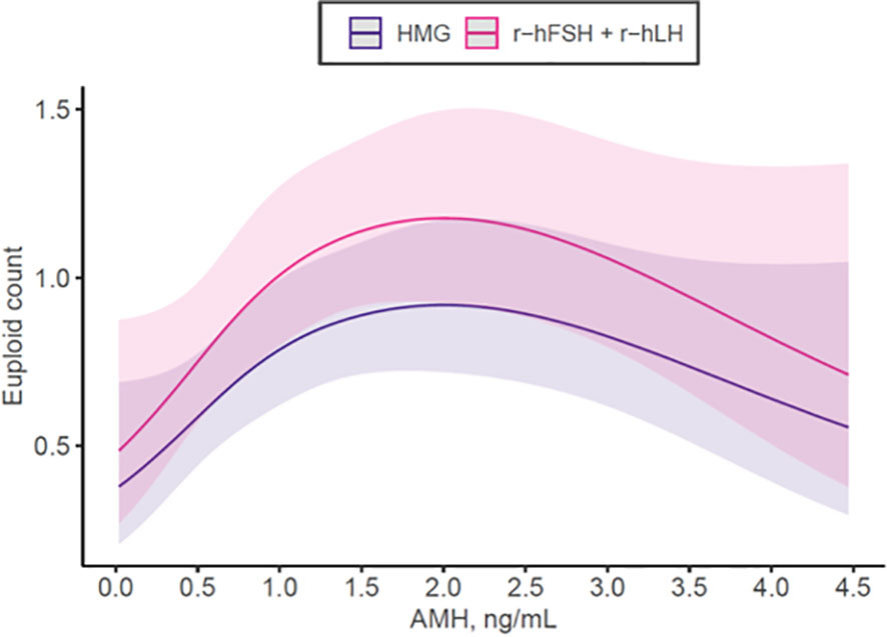
Number of euploid embryos obtained depending on Anti-Muellerian-Hormone (AMH) and stimulation medication used according to multivariable regression model adjusting for AMH, basal FSH, starting dose, female age, BMI, total stimulation duration in the matched cohort.

For mature oocyte count, r-hFSH+r-hLH was associated with a 12% higher yield compared to HMG (IRR 1.12, 95% CI 1.04-1.21, p=0.003) after adjusting for confounders (**Table 5**; **Figure 2**). The sensitivity analysis looking at the interaction of AMH with gonadotropin type showed the effect is mostly significant for those with normal ovarian reserve (AMH between 1.0-3.5ng/mL, IRR: 1.19, 95% CI: 1.07 to 1.33, P = 0.001) but not for those with low (≤1ng/mL, IR: 1.08, 95% CI: 0.97-1.21, P = 0.180) or high ovarian reserve (>3.5ng/mL, IRR: 1.01, 95% CI: 0.72-1.42, P = 0.932). We investigated euploid blastocyst count after stimulation in the matched cohort and HMG use was associated with a 22% lower yield compared to r-hFSH+r-hLH (IRR 0.78, 95% CI 0.66-0.93, p=0.006) after adjusting for AMH, basal FSH, female age, dose, body mass index (**Table 6**; **Figure 3**).

**Table 5:** Mixed-effects negative binomial regression analysis for factors associated with metaphase II oocyte count, matched cohort.

Variables	Incidence rate Ratios	95% CI	P*
Gonadotropin type			
• HMG	Reference		
• r-hFSH+r-hLH	1.12	1.05 – 1.20	**0.001**
AMH	1.15	1.10 – 1.19	**<0.001**
FSH basal	0.96	0.95 – 0.97	**<0.001**
AFC basal	1.07	1.06 – 1.08	**<0.001**
Gonadotropin starting dose			
• [300 IU]	Reference		
• [375 IU]	1.20	1.02 – 1.42	**0.027**
• [450 IU]	1.19	1.05 – 1.34	**0.005**
Female age, SART			
• <35	Reference		
• 35-37	1.00	0.89 – 1.13	0.975
• 38-40	0.91	0.81 – 1.01	0.072
• 41-42	0.87	0.77 – 0.98	**0.018**
• >42	0.86	0.77 – 0.97	**0.010**
BMI category			
• ≤25 kg/m^2^	Reference		
• >25 to ≤30 kg/m^2^	0.97	0.89 – 1.06	0.544
• ≥30kg/m^2^	0.96	0.87 – 1.05	0.350
Stim days (n)	1.09	1.07 – 1.12	**<0.001**

*Mixed effects multivariable negative binomial regression.

AMH, Anti-Mullerian-Hormone; BMI, Body Mass Index; SART, Society for Assisted Reproductive Technology; Gn, Gonadotropin; E2, estradiol; P4, progesterone; COC, Cumulus oocyte complex; MII, metaphase II oocyte; HMG, human menopausal gonadotropin.

Bold values are significant.

**Table 6:** Mixed-effects negative binomial regression analysis for factors associated with euploid count, matched cohort.

Variables	Incidence rate ratios	95% CI	P**
Gonadotropin type			
•r-hFSH+r-hLH	Reference		
•HMG	0.78	0.66 – 0.93	**0.006**
AMH [1st degree]*	1.47	0.64 – 3.35	0.361
AMH [2nd degree]*	0.27	0.10 – 0.70	**0.012**
FSH basal	0.99	0.95 – 1.02	0.382
AFC basal [1st degree] †	5.34	2.21 – 13.08	**<0.001**
AFC basal [2nd degree] †	2.57	1.33 – 4.86	**0.006**
Gonadotropin starting dose			
· [300 IU]	Reference		
· [375 IU]	0.92	0.66 – 1.28	0.638
· [450 IU]	0.73	0.57 – 0.94	**0.014**
Female age, SART			
· <35	Reference		
· 35-37	0.75	0.60 – 0.94	**0.014**
· 38-40	0.52	0.41 – 0.65	**<0.001**
· 41-42	0.43	0.32 – 0.56	**<0.001**
· >42	0.13	0.08 – 0.19	**<0.001**
BMI			
BMI [1st degree]‡	2.13	1.01 – 4.58	**0.048**
BMI [2nd degree] ‡	1.5	0.89 – 2.51	0.122
Stim days (n)	1.08	1.02 – 1.14	**0.006**

*Restricted cubic splines with knot placed at 1.04.

†Restricted cubic splines with knot placed at 8.

**‡** Restricted cubic splines with knot placed at 28.3.

** Mixed effects multivariable negative binomial regression.

Bold values are significant.

## Discussion

The present study demonstrates that in women, who received a stimulation dosage of at least 300 IU, the use of r-hFSH+r-hLH results in a significantly higher mature oocyte yield compared to human menopausal gonadotropin (HMG). This effect is mostly significant for patients with normal ovarian reserve, but not for those with low or high ovarian reserve, measured by AMH. Whereas the number of mature oocytes was higher, the maturation rates per retrieved oocyte were similar in both groups. Therefore, the observed difference is likely due to increased follicular recruitment rather than improved maturation efficiency. However, r-hFSH+r-hLH was associated with an increase in the euploid count. These findings provide valuable insights into optimizing stimulation protocols for this challenging patient population.

It is well established that recombinant FSH (r-hFSH) is associated with a higher oocyte yield compared to HMG, as previously demonstrated in randomized controlled trials (12). Prior studies have suggested that HMG produces fewer oocytes due to its LH activity, which is derived from human chorionic gonadotropin (hCG). These studies have primarily focused on the LH-like activity of hCG, attributing a more sustained stimulation of theca cells to this characteristic.

However, the findings of the present study challenge these previously established notions. Unlike earlier comparisons between HMG-HP and FSH monotherapy, this study directly compares r-hFSH+r-hLH with HMG. Our results suggest that the superior performance of r-hFSH+r-hLH in terms of mature oocyte number may be attributed not solely to LH activity, but rather to the distinct composition and isoform profile of FSH. Additionally, it is important to recognize that LH-like activity, such as that conferred by hCG in HMG-HP preparations, may not replicate the physiological role of LH and has been associated with pro-apoptotic effects in granulosa cells. These findings underscore the need for further investigation into both the qualitative differences in FSH isoforms and the divergent biological effects of true LH versus LH-like activity.

A longstanding debate in reproductive medicine concerns the contribution of LH activity to ovarian response during controlled ovarian stimulation. The primary objective of maximizing oocyte yield is to increase the number of viable embryos available for transfer and cryopreservation, ultimately improving the likelihood of live birth. Particularly in women requiring high dosage stimulation, LH supplementation has been suggested to enhance FSH receptor expression, improve follicular recruitment, and reduce granulosa cell apoptosis (5, 6). A meta-analysis by Lehert et al. (4) demonstrated that in women with poor ovarian response, significantly more oocytes were retrieved with r-hFSH/r-hLH compared to r-hFSH alone (weighted mean difference +0.75 oocytes; 95% CI 0.14, 1.36), supporting the beneficial role of LH co-treatment. However, our study provides a distinct comparison by evaluating r-hFSH+r-hLH against HMG, where the LH activity primarily originates from hCG (7). Notably, despite a lower oocyte yield in the HMG group compared to the r-hFSH+r-hLH group, systemic FSH levels at the time of final oocyte maturation were significantly higher in the HMG group (HMG group: 25.7 (21.2 to 31.3) IU vs r-hFSH+r-hLH group: 23.2 (19.4 to 28.4) IU, p<0.001). This finding can be explained by differences in FSH isoform composition and elimination kinetics between gonadotropin preparations, rather than the LH activity. FSH exists in multiple isoforms with varying glycosylation patterns, influencing their acidity, receptor-binding affinity, and *in vivo* half-life. More acidic FSH isoforms exhibit lower receptor-binding affinity but prolonged half-life, leading to sustained biological activity, whereas less acidic isoforms demonstrate higher receptor affinity but shorter half-life. Recombinant FSH predominantly consists of less acidic isoforms, facilitating rapid receptor activation and clearance. In contrast, HMG-derived FSH contains a broader range of isoforms, including more acidic variants, contributing to an extended circulatory half-life and sustained FSH activity (13). These pharmacokinetic differences may influence ovarian response, particularly in suboptimal responders requiring optimized gonadotropin support. The higher receptor-binding affinity of recombinant FSH may explain the significantly higher oocyte yield observed in the r-hFSH+r-hLH group (13).

A major strength of this study is the large sample size, with a total of 1,286 patients analyzed. After optimal pair matching, 526 patients per group were included, ensuring comparable baseline characteristics, including age, BMI, AMH, basal estradiol, basal FSH, and starting gonadotropin dose. This rigorous methodological approach enhances the reliability of our findings and underscores the importance of gonadotropin selection. Additionally, a key strength of this study is the detailed measurement of systemic FSH levels during stimulation, a factor often overlooked in prior research (14). Understanding the pharmacokinetics of gonadotropin preparations is crucial for refining stimulation protocols and optimizing outcomes. As a retrospective study, causality cannot be confirmed, and despite rigorous matching, unmeasured variables such as individual gonadotropin sensitivity may have influenced the results. A limitation of this study is that pregnancy and live-birth outcomes were not assessed. As the primary objective was to evaluate ovarian response, mature oocyte yield and euploid yield, clinical downstream outcomes such as cumulative pregnancy rate were not within the scope of our research and would be difficult to assess as cumulative pregnancy rate can only be evaluated after the transfer of all euploid embryos from the cohort. This fact limits the ability to determine the full clinical impact of the observed differences in mature oocyte yield. Recent evidence points towards an impact of genetic polymorphisms on oocyte yield and maturation outcomes and suggests that some genetic variants may alter the requirement for LH supplementation during ovarian stimulation, with some individuals achieving adequate response at lower LH doses or without LH addition. We did not assess genetic polymorphism in our study, which could be considered as a further limitation of this analysis.

## Conclusion

This study demonstrates that stimulation with r-hFSH+r-hLH yields significantly higher mature oocyte counts compared to HMG in women in women who received 300 IU or more to eliminate gonadotropin dose as a confounding factor. Whereas the maturation rates were similar between the groups, a higher euploid count was found in the r-hFSH+r-hLH group. Clinicians could consider integrating these insights into individualized stimulation strategies to optimize outcomes in this patient population. Given the pivotal role of mature oocyte number in IVF success, these findings warrant further validation through future randomized controlled trials which would provide the highest level of evidence to re-enforce our findings.

## Data Availability

The original contributions presented in the study are included in the article/**Supplementary Material**. Further inquiries can be directed to the corresponding author.
