# Supplementary material for: Optimizing mature oocyte yield in IVF: clinical comparison of r-hFSH+r-hLH and HMG in women with a stimulation dosage of at least 300 IU of gonadotropins
Source: Front Endocrinol (Lausanne). 2026 Jan 29;17:1696657. doi: 10.3389/fendo.2026.1696657 (PMC12893992; doi:10.3389/fendo.2026.1696657)
Supplement: Supplementary file 1 [file SupplementaryFile1.docx]

**Supplementary figure S1**. Mean differences of covariates before and after the matching.


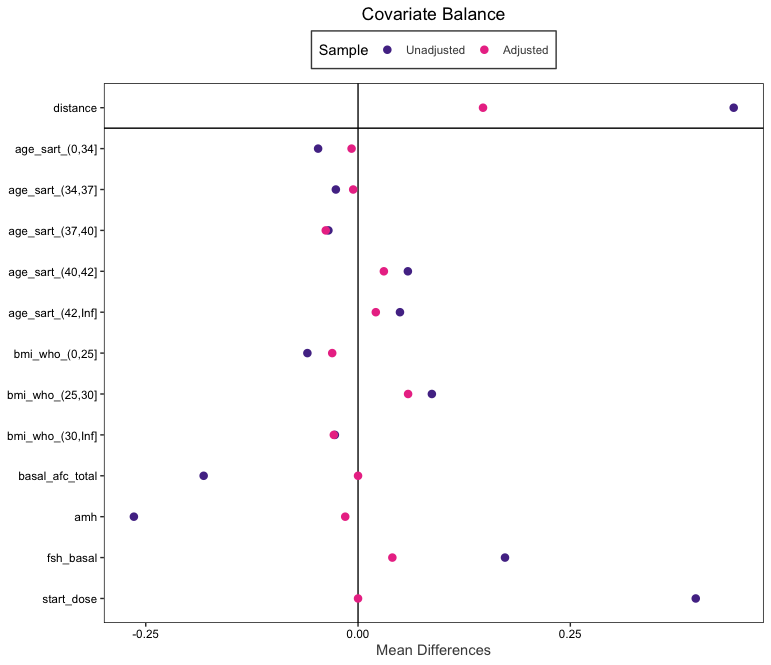


BMI: Body Mass Index; WHO: World Health Organization; SART: Society for Assisted Reproductive Technology; AFC: Antral follicle count; start dose: starting Gonadotropin dose; FSH basal: FSH level at stimulation start

**Supplementary figure S2**. Distributional balance of covariates after the matching.


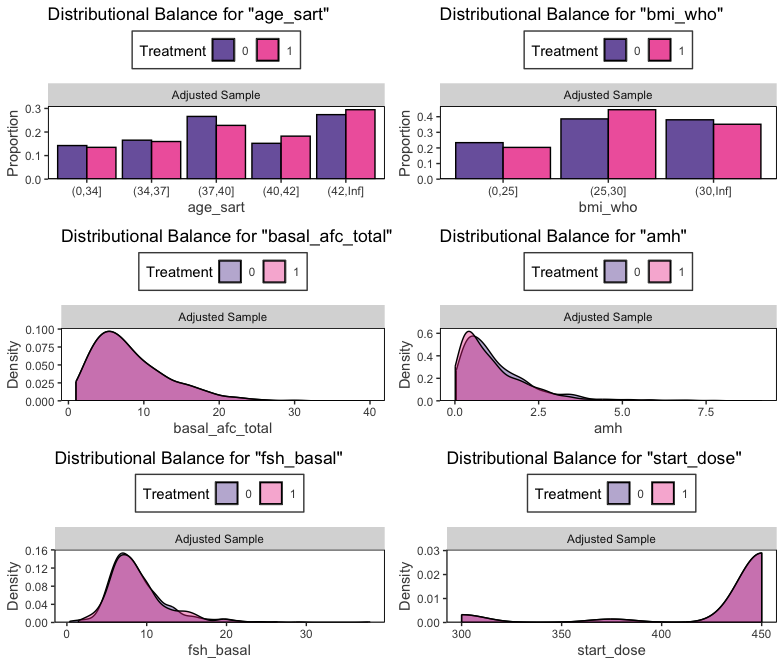


BMI: Body Mass Index; WHO: World Health Organization; SART: Society for Assisted Reproductive Technology; AFC: Antral follicle count; start dose: starting Gonadotropin dose

FSH basal: FSH level at stimulation start
